# Deadly Combination of Formic and Sulfuric Acid: 2 Cases of Carbon Monoxide Intoxication

**DOI:** 10.1097/FTD.0000000000001410

**Published:** 2025-12-02

**Authors:** Corine Bethlehem, Nanda M. van Dam, Linda G. W. Franken, Dieuwertje van den Bogart, Marika Wind, Birgit C. P. Koch

**Affiliations:** * Erasmus MC, University Medical Center Rotterdam, Department of Hospital Pharmacy, the Netherlands;; † Department of Hospital Pharmacology and Clinical Pharmacology, Amsterdam UMC, Location University of Amsterdam, Amsterdam, the Netherlands;; ‡ Forensic Physicians Rotterdam (FARR), the Netherlands; and; § Public Health Region Service (GGD) Region Utrecht, the Netherlands.

**Keywords:** carbon monoxide, formic acid, sulfuric acid, intoxication

## Abstract

**Background:**

Carbon monoxide (CO) poisoning is associated with high morbidity and mortality rates. Although accidental inhalation is the most common cause, suicide through CO from a mixture of formic and sulfuric acids is extremely rare and poses unique risks to first responders.

**Case presentations:**

Two male individuals died in their vehicles after mixing formic and sulfuric acid to generate CO. Both cases showed characteristic cherry-red or pink livor mortis and high postmortem carboxyhemoglobin levels (73% and 85%, respectively). In both scenes, labeled acid containers and enclosed spaces were present, with 1 case noting an unusual odor.

**Conclusions:**

Chemically induced CO poisoning, although rare, requires heightened awareness among first responders because of the danger of undetected exposure. Key indicators include unusual livor mortis, enclosed or taped-off spaces, chemical smells, and the presence of acid containers. Postmortem toxicology is crucial for confirming a diagnosis and understanding the cause of death.

## BACKGROUND

Every year, approximately 10 deaths and 200 hospitalizations due to carbon monoxide (CO) poisoning were recorded in the Netherlands.^1^ CO is a highly toxic gas because its affinity for hemoglobin is 200 times greater than that of oxygen. As a result, CO prevents oxygen from binding to hemoglobin, forming carboxyhemoglobin (COHb) and reducing oxygen release in peripheral tissues. The initial symptoms of CO poisoning include headaches, dizziness, dyspnea, and nausea. Increased exposure may result in hypotension, confusion, seizures, cardiac arrhythmias, coma, and eventually death.^2^

Most deaths due to CO poisoning are caused by accidental CO inhalation. Suicide by CO is much rarer, and if CO is used as a suicide method, the source of CO is almost always incomplete combustion of carbon.^3^ A recent study conducted in the Czech Republic examined 495 CO suicide attempts over the past 60 years. It was found that 66% were caused by coal gas, and the remaining 34% by burning of other types of fuel, such as vehicle exhaust fumes, gas, and wood.^2^

CO intoxication, originating from the combination of formic acid and sulfuric acid, is extremely rare. An overview of 9 case reports that used a mixture of the 2 acids is summarized in the Table 1.^4–10^ In this article, we describe 2 cases of CO suicide due to gases formed after mixing formic and sulfuric acids. We highlight the potential dangers to first responders, considering that CO is odorless, along with the importance of postmortem forensic blood analysis.

**TABLE 1.** Overview of Case Reports Describing the Use of a Mixture of Sulfuric Acid and Formic Acid to Produce CO

	Sex	Age (yr)	Did Patient Die?	Suicide or Accident?	COHb Concentration (pm*/Alive; Estimated PMI)	Smell at Place Where Body was Found	Place Where Body was Found	Livor Mortis	Chemical Substances Found?
Prahlow and Doyle^4^	Male	21	Yes	Suicide	64% pm (unknown)	Chemical odor	Bedroom closet taped shut	Bright red lividity	Home-made device, later discovered to be formic acid and sulfuric acid
Yang et al^5^	Male	26	Yes	Suicide	Unknown	Pungent odor	Bedroom	Unknown	Three beakers, containing formic acid (98%–100%) and sulfuric acid (95%–98%)
	Male	53	No	Accident	45.8% alive			Unknown	
	Female	53	No	Accident	23% alive			Unknown	
Lin and Dunn^6^	Male	26	Yes	Suicide	85% pm (24 h)	Chemical odor	Car	Bright pink–red lividity	Empty bottle of sulfuric acid-based drain opener, another empty bottle with an illegible label, and an invoice from a chemical supply company for 950 mL of formic acid
Bakovic et al^7^	Male	22	Yes	Suicide	30% pm (unknown)	Penetrating chemical smell	Locked bathroom	Cherry-pink livor mortis	An open plastic 5-L canister, containing approximately 1.1 L of liquid, was found in front of the house. The canister had a label “formic acid, 85%, made by CIDA SpA Italy.” Inside the bathroom, there was an empty plastic 5-L canister with no label, together with a 10-L bucket filled by half with unknown fluid
Kreutz et al^8^	Male	44	Yes	Suicide	83% pm (24 h)	“Ammonia bleach” odor	Vehicle	Cherry-red lividity	Beside the bowl were 2 empty bottles of commercially available sulfuric acid-based drain cleaner, 1 nearly empty bottle of 95% concentrated formic acid, rubber gloves, 1 unopened box of baking soda (sodium bicarbonate), and a 32 ounce measuring cup
Aaron Schneir et al^9^	Male	31	No	Suicide	36.8%, 3.4%** alive	Faint odor of a chemical	Motel room, closed bathroom with vent taped shut	Unknown	Unlabeled five-gallon bucket with a hose inserted through the lit, later they found a total of 5 separate containers of sulfuric acid, one of which was opened and appeared nearly full. The unlabeled 5-gallon bucket with attached hose was approximately half-full of clear fluid
Lewis et al^10^	Male	21	Yes	Suicide	27.4% alive	Unknown	Residence	Unknown	A large bowl containing an unknown solution, which was later identified as a mixture of sulfuric acid and formic acid.

PMI, Post Mortem Interval, ** the second value (3.4%) was measured 4 h after the first measurement.

## CASE PRESENTATIONS

### Case 1

A 35-year-old man was found dead early in the morning in his car, which was parked in the parking lot of a company where he worked. The man was discovered by colleagues who did not know him well. The next of kin, friends, and acquaintances were unknown, he had reportedly worked as a chemist in France in the past.

The deceased sat in the driver's seat, which was tilted slightly backward. The man's arms hung beside his body and the head tilted slightly to the right. The patient wore a plastic glove in the right hand. Warnings were affixed to the car windows, displaying a skull symbol and the text “carbon monoxide.” The passenger seat was a sink with liquid. Two empty plastic one-liter bottles containing formic acid and sulfuric acid were placed in front of the passenger seat according to the labels.

**FIGURE 1.**
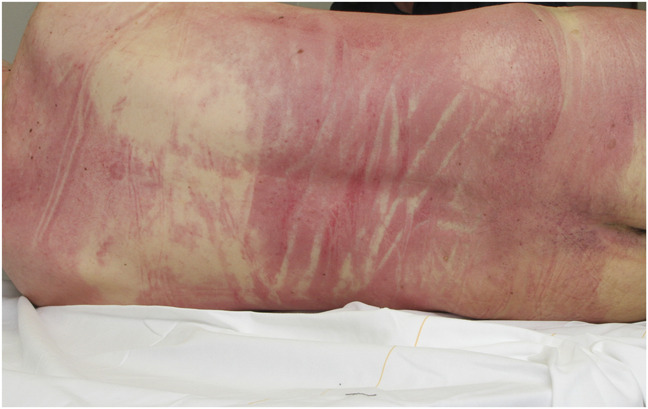
Case 1: Cherry-red livor mortis on the back.

Because of the suspicion of CO intoxication—based on the warnings on the windows, the bottles found, and visible cherry-red livor mortis of the left hand—the fire brigade was called. After examining the car and removing the liquid, his body was evacuated. Inspection and further examination of the body were performed in the mortuary of a nearby hospital. Scattered over the body (right side of the face, back, back of the arms and thighs, hands, and lower legs), cherry-red livor mortis was found (Fig. 1), according to the position of the body, as found in a car. The livor mortis remained blanchable. There was a generalized body rigidity that could have been broken. The body temperature and ambient temperature, measured in the morgue, were 32.2 and 16°C, respectively, with an estimated time postmortem interval of 11 hours according to the method of Henssge^11^ and an estimated body weight of 80 kg. No injuries were observed on the body.

Postmortem toxicological analysis of postmortem blood samples revealed a COHb level of 73%. Furthermore, the blood contained fluoxetine, bromazepam, and dipyridamole, although no concentrations were measured, which were presumed to be within the therapeutic range and not within the toxic range. This could indicate a depressive disorder. The blood alcohol level was circa 0,7 promille.

After the postmortem investigation, it was concluded that the man had committed suicide by mixing formic acid (85%) and sulfuric acid (95%) while sitting in a car, resulting in a high concentration of CO in the closed car. The patient died of CO poisoning.

### Case 2

A 19-year-old man was found dead in a car in a parking lot. He was reported missing by his mother the day after he was last seen around midnight the day before.

The man sat in the driver's seat of the car, leaning forward with his head lowered against the steering wheel. The paramedic who opened the car door immediately noticed a pink livor mortis on the arms. The body was completely rigid. An odor similar to that of rotten eggs was observed.

Owing to the suspicion of the presence of CO based on the pink-colored livor mortis spots, the fire brigade arrived at the scene. From a window that had broken immediately before initial assistance, a heat source was detected in the car. A bucket covered with clothing containing an acidic and heat-producing liquid was found in the back seat. The CO content of the air above the bucket, measured 2 hours after encountering the body, was 54 ppm, whereas the normal CO content in open air was 5 ppm.^12^ Two half-filled liter bottles containing formic acid and sulfuric acid according to the label were found in the car.

After examining the car and neutralizing the liquid, the man's body was safely evacuated.

Fixed and pink-colored livor mortis spots were observed scattered over the abdomen, forearms, and hands. This was in accordance with the position of the body. Blue livor mortis spots were visible on the anterior neck. Generalized rigor mortis was set so strongly that it was impossible to relax the muscles. The temperatures of the body and environment were not measured because the presence of an active heat source could have interfered with the interpretation of the results. No signs of decomposition were observed. Postmortem toxicological screening showed COHb levels of 85%, in addition to a blood alcohol concentration of circa 1,2 promille and the presence of a low concentration of paracetamol and cocaine and its metabolites in the femoral blood.

## DISCUSSION

Between 2018 and 2024, an average of 67 CO poisoning incidents per year were registered at the Dutch Poisons Information Center. These numbers represent accidents with CO gas, such as in the case of a leaking boiler system. Of the registered incidents, only a small minority depicted suicides, and sporadically, there were suicide cases that used a mixture of formic acid and sulfuric acid to form CO.^3^

In both cases, CO is produced by mixing formic and sulfuric acids. Sulfuric acid is a hygroscopic substance that forms bonds with water. Therefore, when sulfuric acid is mixed with formic acid, formic acid disintegrates, resulting in a mixture of sulfuric acid, water, and CO (Equation 1). The formed CO binds to hemoglobin and probably causes the death of both individuals owing to its hypoxic effects.

(1) $$CH_{2}O_{2}+H_{2}O_{4}S\;\to CO+H_{2}O_{4}S*\;H_{2}O$$

Equation 1: How formic acid and sulfuric acid can create CO.

Therefore, according to the Dutch Poison Information Center (DPIC), these types of toxicity are unusual. This indicates that the conditions under which the deceased or patients were found were relatively unknown. In addition, cases may differ in the clues leading to diagnosis, varying from smell to warning, to the visibility of chemical compounds.

Therefore, it is important for first-aid responders to be aware of this rare CO suicide method due to the dangers of CO gas. In the first case, the victim posted warning signs in his vehicle, causing the medical examiner to take appropriate measures. However, the second case illustrates that this is not the case in every instance; therefore, first-aid responders should take extra care when they see any indications of CO intoxication. A case report by CC Yang et al stated that CO can contaminate that can affect first responders.

As previously stated, the primary objective of this case report was to call attention to the potential hazards for first-aid responders, in addition to the additional value of postmortem toxicological screening. Therefore, we attempted to identify indications for chemical CO intoxication.

## INDICATORS

A previous study conducted by Nielsen et al^13^ showed that cherry-red livor mortis is typical of CO intoxication. Given that the formed COHb has a spectrum comparable with that of oxyhemoglobin, red livores can also occur in cases of hypothermia or cyanide poisoning because of the resaturation of hemoglobin with O_2_.^14^ The circumstances under which the deceased were found in these cases were highly indicative of CO poisoning. In the present case, cherry-red and pink livor mortis were observed. These livor mortis are in line with the found literature, as summarized in Table 1. Interestingly, in the second case, blue-colored livor mortis was reported. This can be explained by cyanosis resulting from CO intoxication. Livor mortis, mainly cherry-red or pink, can be an important first indication of CO intoxication. Therefore, when livor mortis is noticed, the first responders need to be cautious.

It is noteworthy that, as summarized in the table, many of the deceased were young. Our case is no exception. It is difficult to distinguish exactly why men are in the majority here; possibly because men are more represented in chemistry-related occupations. Of course, it could be that because of the extremely small number of suicides using the mixture of formic acid and sulfuric acid, the men–women ratio is incorrect.

Both the deceased were found in vehicles, which are well-known places for suicide by CO. However, this is not necessarily the case when CO is formed from a mixture of formic and sulfuric acids. In the literature, more cases have been reported in enclosed spaces than in vehicles. In most cases, ventilation was prevented in enclosed spaces. Thus, extra care is required when an enclosed space is encountered, such as when the doors or vents are closed.

Although CO is odorless, the paramedics in the second case noticed a smell similar to that of rotten eggs. This has not yet been described in the literature as a pungent smell is mostly identified at the location where the body is found. This pungent smell is often attributed to formic acid, which has a strong chemical odor. The rotten egg smell is typical of hydrogen sulfide; however, this is not an expected substance obtained from the reaction of formic acid and sulfuric acid. It could be that sulfuric acid was contaminated and led to a similar smell that way, or maybe the paramedics wrongly identified the smell. Because sulfuric acid is odorless, CO is odorless, and formic acid has a distinctive chemical smell, the pungent smell at the crime scene implies that a mixture of formic acid and sulfuric acid produces CO.

In both cases, bottles were clearly labeled by the manufacturer with formic acid and sulfuric acid. We found that finding bottles or cannisters is quite common for chemically formed CO toxicity. Therefore, the presence of bottles with labels, unknown clear liquids, or sulfuric acid-based drain cleaners may suggest CO poisoning.

## ANALYSIS

Suspected CO intoxication can be confirmed by postmortem COHb analysis. The Erasmus Medical Center routinely performs postmortem toxicological screening as part of the postmortem external examinations. This toxicological investigation involved screening of femoral blood using liquid chromatography–mass spectrometry (LC-MS/MS), blood alcohol concentration, and Drug of Abuse in urine. When a criminal offense is suspected, toxicological analysis is conducted by the Dutch forensic institution; in other cases, screening is also performed by the laboratory of a selected group of hospitals. In addition to routine screening, a COHb test can be performed when indicated. COHb is formed when hemoglobin is bound to CO and can be measured as a percentage of the total hemoglobin. Most deaths due to CO intoxication are associated with COHb levels >40%.^15^ In these cases, COHb analysis confirmed CO poisoning, with COHb concentrations of 73% and 85%. Spectrophotometric analysis is also accurate for postmortem blood samples.^16^ In the literature, the COHb levels of deceased vary from 30% to 85%. Therefore, our observations are consistent with existing literature.

In conclusion, we described 2 patients who died due to CO intoxication after mixing formic and sulfuric acids. Owing to its hazardous nature, raising awareness regarding this type of chemically induced CO poisoning is crucial. Early recognition of these signs can help prevent exposure and reduce the risk of harm. Therefore, we identified numerous indicators of CO intoxication that can help first responders estimate whether extra care is needed when approaching a victim. These indicators included pink or cherry-red livores, enclosed spaces (eventually taped down), and a chemical smell (although CO itself is odorless) and the visibility of any substance at the location of the body.

Postmortem blood analysis is essential to determine the cause of death. In addition, postmortem analysis can create awareness and provide families with a deceased closure. In the case of suicide or an accident, CO intoxication can be either confirmed or excluded by toxicological screening.
